# Hepatitis B Vaccination Status and Needlestick Injuries Among Healthcare Workers in Syria

**DOI:** 10.4103/0974-777X.59247

**Published:** 2010

**Authors:** Rabi Yacoub, Radwan Al Ali, Ghamez Moukeh, Ayham Lahdo, Yaser Mouhammad, Mahmood Nasser

**Affiliations:** University at Buffalo, NY, USA; 1 Department of Internal Medicine, Aleppo University Hospital, Aleppo, Syria

**Keywords:** Needlestick injuries, Hepatitis B infection, Healthcare workers

## Abstract

**Background:**

Although a majority of countries in the Middle East show intermediate or high endemicity of hepatitis B virus (HBV) infection, which clearly poses a serious public health problem in the region, the situation in the Republic of Syria remains unclear. The aim of this study is to determine the hepatitis B vaccination status, to assess the number of vaccinations administered, and to estimate the annual incidence of needlestick injuries (NSIs) among healthcare workers (HCWs) in Aleppo University hospitals.

**Materials and Methods:**

A cross-sectional design with a survey questionnaire was used for exploring details of NSIs during 2008, hepatitis B vaccination status, and HBV infection among a random stratified sample of HCWs in three tertiary hospitals in Aleppo (n = 321).

**Results:**

Two hundred and forty-six (76.6%) HCWs had sustained at least one NSI during 2008. Nine (2.8%) had HBV chronic infection and 75 HCWs (23.4%) were never vaccinated. Anesthesiology technicians had the greatest exposure risk when compared to office workers [OR = 16,95% CI (2.55-100), *P* < 0.01], doctors [OR = 10,95% CI (2.1 47.57), *P* < 0.01], and nurses [OR = 6.75,95% CI (1.56-29.03), *P* = 0.01]. HCWs under 25 and between the age of 25 and 35 years were at increased risk for NSI when compared to HCWs older than 45 years [OR = 3.12,95% CI (1.19-8.19), *P* = 0.02] and [OR = 3.05,95% CI (1.42-6.57), *P* < 0.01], respectively.

**Conclusion:**

HCWs at Aleppo University hospitals are frequently exposed to blood-borne infections. Precautions and protection from NSIs are important in preventing infection of HCWs. Education about the transmission of blood-borne infections, vaccination, and post-exposure prophylaxis must be implemented and strictly monitored.

## INTRODUCTION

Healthcare workers (HCWs) who use and are exposed to needles are at an increased risk of needlestick injuries (NSIs). Data show that more than 20 diseases have been perceived to be transmitted to HCWs by NSI,[1] resulting in the increased risk of having blood-borne infections such as hepatitis B virus (HBV), hepatitis C virus (HCV), and human immunodeficiency virus (HIV), with HBV being the most common blood-borne pathogen that poses an occupational risk to HCWs.[2,3]

Occupational Safety and Health Administration (OSHA) estimates that 5.6 million American HCWs are at risk of occupational exposure to blood-borne pathogens, and it has also estimated that 800,000 NSIs occur annually in hospitals in the United States. Worldwide it has been estimated that in 2000 alone, percutaneous injuries led to 16,000 cases of hepatitis C, 66,000 cases of hepatitis B, and 1,000 cases of HIV.[4]

The incidence of HBV infection following NSI has been reported to be high among unvaccinated medical staff. Therefore, vaccination against HBV should be given to any person who performs tasks involving contact with blood, blood-contaminated body fluids, other body fluids, and sharps.[5,6] The vaccine should always be administered by the intramuscular route in the deltoid muscle with a needle 1-1.5 inches long. Hepatitis B vaccine can be administered at the same time as other vaccines with no interference of antibody response to other vaccines.[7] If the vaccination series is interrupted after the first dose, the second dose should be administered as soon as possible. The second and third doses should be separated by an interval of at least two months. If only the third dose is delayed, it could be administered when convenient.[8]

Needlestick injuries may occur when HCWs dispose of needles, collect and dispose of materials used during patient care procedures, administer injections, draw blood, or handle trash or dirty linens where needles have been inappropriately discarded. The incidence commonly occurs when workers try to do several tasks at the same time.

Although a majority of countries in the Middle East show intermediate or high endemicity of HBV infection, which clearly poses a serious public health problem in the region, the situation in the Republic of Syria, remains unclear.[9,10]

At present it is assumed that a majority of HBV infections occur through childhood and perinatal transmission, but results have been conflicting and information on the role of sexual behavior and unsafe injection practices remains limited. Methods that are commonly employed for preventing HBV infection are universal methods (i.e., infant immunization), targeted (high-risk groups), routine HbsAg screening, and implementing safe injection practices.[9] Universal vaccination has been introduced through the Expanded Program on Immunization (EPI) in Syria, since 1991, which has resulted in a coverage rate of 76%. With regard to occupational HBV transmission, substantial work has been done in the last decade through a partnership program with WHO (SIGN Network Focus Project) and the Syrian Ministry of Health, aimed at improving injection safety of immunization among healthcare workers, and resulting in Syria being considered one of the safest countries in the WHO Eastern Mediterranean Region.[11] Hospitals that still do not implement injection safety interventions, however, yet face major risks of nosocomial transmission of blood-borne pathogens.

In this study we aim to define prevention priorities on the basis of collection and analysis of the institution's injury data, which are the key to identifying injury patterns, and then implementing an effective abatement plan. We also need to emphasize that NSIs are not just ‘part of the job’ and that all NSIs should be reported.

## MATERIALS AND METHODS

This cross-sectional study was conducted in 2008 and approved by the Aleppo University Human Research and Ethical Committee. Data were collected from three tertiary University Hospitals, which are the most important public hospitals in the city of Aleppo, with its 2,000,000 residents. These hospitals include a staff of 1052 nurses, 541 doctors, 658 housekeeping workers, 297 laboratory technicians, 270 anesthesiology technicians, 301 dialysis technicians, and 93 office workers practising on medical floors. The sample size was calculated for a power of at least 80%, 5% margin of error, confidence interval (CI) of 95%, and due to the high incidence of NSIs in the previous research,[2,9–11] the expected proportion for response was considered as 65%. Based on these parameters, the sample size was calculated as at least 316 out of 3212 healthcare workers.

Subjects were selected from each group using a computerized randomization technique based on house staff medical record numbers, with equal probabilities, using a stratified random sampling method. Four hundred invitations were sent for HCWs who worked for at least one year in one of the hospitals, and in total, 321 HCWs responded (80.25 %). After taking the informed consent, the subjects were asked to answer an interviewer-administrated self-developed questionnaire [Table 1], which included demographics and information about years of working in the hospital, department/place of work, and history of NSI and Hepatitis B vaccination.

**Table 1:** The Questionnaire

Part One: To be filled by the researcher in the presence of the HCW				
Name				
Age				
Subject number				
Work type				
Nurse				
Doctor				
Housekeeping worker				
Laboratory technician				
Anesthesiology technician				
Dialysis technician				
Office worker				
Work duration in medical field (in months)				
NSI	Yes	O	No	O
History of NSI in the last 12 months				
Number of NSIs in the last 12 months				
Please describe the situation when the NSI happened				
Vaccinations against HBV (date)	Yes	O	No	O
1^st^ dose				
2^nd^ dose				
3^rd^ dose				
Booster				
Last known anti HBV Ab (date and titer)
Current laboratory results	Positive	O	Negative	O
HBs Ag				
Anti HBV Ab titer				
Anti HBC Ab				

**Part two: Medical records (To be filled by the researcher)**				

NSIs recorded	Yes	O	No	O
History of NSI in the last 12 months				
Number of NSI in the last 12 months				
Please describe the situation when the NSI happened				
Vaccinations against HBV (date)	Yes	O	No	O
1^st^ dose				
2^nd^ dose				
3^rd^ dose				
Booster				
Laboratory results (date)	Positive	O	Negative	O
HBs Ag				
Anti HBV Ab titer				
Anti HBC Ab				
Note:				

NSI = Needlestick injuries; HBV = Hepatitis B virus; HBC = Hepatitis B Core; HBs

Ag = Hepatitis B Surface Antigen

We defined NSI as an injury with a contaminated needle, whether it was used for intravenous introduction of medications, drawing blood, or wound suture. Data from self-reported injuries probably underestimate the severity of the problem because many workers do not report their injuries. This makes it difficult to know exactly how serious the problem is or how well prevention programs work. Furthermore, depending only on the questionnaire may also raise the chance of having a high rate of recall bias. Therefore, data were gathered from both employees and hospital records (NSI reports and the medical record of each employee), including their vaccination charts and previous laboratory results.

At the time of interview, each person was interviewed face-to-face, a blood sample was drawn for new laboratory tests, and a questionnaire regarding their previous NSIs was completed. Hepatitis B surface antigen was tested using FirstVue™ HBsAg rapid test (First Diagnostic LLC. USA), and anti-HBs antibodies were tested using WB-2896™ HBsAb ELISA test (Wantai Biological Pharmacy Enterprise Co., Ltd. China).

The approved schedule for hepatitis B vaccination was the usage of 1.0 mL Engerix-B (GlaxoSmithKline, UK), at 01, and 6 months. We defined the HCW as completely vaccinated if he/she had followed the Center for Disease Control and Prevention (CDC) vaccination criteria, and as incompletely vaccinated if he/she had got at least one dose of vaccination, but did not follow the CDC criteria for complete vaccination.[8]

Data were analyzed using SPSS® fifteenth edition statistical program (Statistical Package for the Social Sciences, SPSS Inc. IL, USA). Chi square test was used for bivariate analysis of the association between categorical variables with main outcomes (e.g., NSI). Multivariate logistic regression was used to assess the exposure risk by type of work, age group, and work duration as dependent confounding risk factors, with the office workers group considered as a reference group in calculating occupational risk, and age of >45 years as a reference group when calculating the risk of exposure among age groups. The regression was done using a stepwise, backward selection procedure; this approach was used since it had the capability of retaining important confounding variables, potentially resulting in a rich model.[12]

## RESULTS

Three hundred and twenty-one workers participated in this study, of whom 32.7% (n = 105) were nurses, 16.8% (n = 54) doctors, 20.6% (n = 66) housekeeping workers, 9.3% (n = 30) laboratory technicians, 9.3% (n = 30) dialysis technicians, 8.4% (n = 27) anesthesiology technicians, and 2.8% (n = 9) office workers [Table 2]. One hundred fifty workers (46.7%) were male.

**Table 2:** Characteristics of the study population

	Occupation						
	
	Nurse (%)	Doctor (%)	Housekeeping worker (%)	Laboratory technician (%)	Anesthesiology technician (%)	Dialysis technician (%)	Office worker (%)
Mean age (±SD)	33.83 ± 5.83	30.11 ± 6.09	32.14 ± 10.48	44.1 ± 10.91	32.56 ± 7.64	30.9 ± 7.41	37.33 ± 10.14
Age (years)							
≤25	6 (11.1)	9 (16.7)	24 (44.4)	0 (0)	6 (11.1)	6 (11.1)	3 (5.6)
26-35	66 (41.5)	36 (22.6)	15 (9.4)	12 (7.5)	12 (7.5)	18 (11.3)	0 (0)
36-45	24 (34.8)	6 (8.7)	18 (26.1)	6 (8.7)	9 (13)	3 (4.3)	3 (4.3)
>45	9 (23.1)	3 (7.7)	9 (23.1)	12 (30.8)	0 (0)	3 (7.7)	3 (7.7)
Gender (Male)	0 (0)	51 (34)	42 (28)	21 (14)	12 (8)	15 (10)	9 (6)
Mean work duration (±SD)	10.54 ± 7.56	4.44 ± 4.72	5.86 ± 7.11	17 ± 9.95	8.56 ± 5.28	9.1 ± 9.41	6.33 ± 3.9
Work duration > years	63 (40.4)	9 (5.8)	21 (13.5)	30 (19.2)	15 (9.6)	12 (7.7)	6 (3.8)

Two hundred forty-six HCWs (76.6%) had at least one NSI during the last year. Nine workers (2.8%) had laboratory evidence of chronic HBV infection; three were housekeeping workers, and six were anesthesiology technicians. All housekeeping workers who had chronic HBV infection were less than 25 years old. Seventy-five HCWs (23.4%) were never vaccinated, 66 (20.6%) received incomplete vaccination, and 180 (56.1%) were completely vaccinated. Vaccination status by work type is shown in Table 3. Even though housekeeping workers are at an increased risk of having NSIs, they hold the lowest vaccination rate resulting in 4.5% of them suffering from HBs chronic infection. When compared to other occupation groups, dialysis technicians had the highest rate of vaccination (90%), which was due to the strict criteria of infection control and prevention precautions applied in dialysis units. The remaining 10% of dialysis technicians were already exposed to HBV and gained active immunity. Doctors were all either completely or partially vaccinated against HBV. Most of the partially vaccinated doctors were residents who had just started their vaccination regimen.

**Table 3:** Vaccination status by work type

Occupation	Never vaccinated (% within work)	Incomplete vaccination (% within work)	Complete vaccination (% within work)	P*
Nurses	9 (8.6)	24 (22.9)	72 (68.6)	0.00
Doctors	0 (0.0)	24 (44.4)	30 (55.6)	0.00
Housekeeping workers	51 (77.3)	3 (4.5)	12 (18.2)	0.00
Laboratory technicians	0 (0.0)	6 (20.0)	24 (80.0)	0.00
Anesthesiology technicians	9 (33.3)	6 (22.2)	12 (44.4)	0.21
Dialysis technicians	3 (10.0)	0 (0.0)	27 (90.0)	0.00
Office workers	3 (33.3)	3 (33.3)	3 (33.3)	0.37

* Compared to all other occupation groups combined

Sixty three HCWs (19.6%) had titers of less than 10 IU/L (nonreactive), 48 (15%) had titers ranging from 10 to 100 IU/L, and 210 (65.4%) had titers of more than 100 IU/L. Table 4 shows anti HBs antibody test results by work type. One hundred sixty nine HCWs (52.4%) had the last dose of immunization more than five years prior to the interview. Of those, nineteen workers (5.9%) had an undetectable level of anti-HBsAb; nine of them previously received three doses of vaccination.

**Table 4:** Anti HBs antibodies test results by work type

Occupation	<10 IU/L (%within work)	10-100 IU/L (%within work)	>100 IU/L (%within work)	P*
Nurses	6 (5.7)	12 (11.4)	87 (82.9)	0.00
Doctors	0 (0.0)	15 (27.8)	39 (72.2)	0.00
Housekeeping workers	39 (59.1)	12 (12.8)	15 (22.7)	0.00
Laboratory technicians	0 (0.0)	3 (10.0)	27 (90.0)	0.00
Anesthesiology technicians	9 (33.3)	6 (22.2)	12 (44.4)	0.05
Dialysis technicians	3 (10.0)	0 (0.0)	27 (90.0)	0.00
Office workers	6 (66.7)	0 (0.0)	3 (33.3)	0.00

* Compared to all other occupation groups combined

Thirty-one HCWs had never been vaccinated and have detectable anti-HBsAb titers, positive anti-HBC antibodies. Seventeen of those were previously diagnosed with acute hepatitis B infection, andthe other fourteen workers never had signs or symptoms of hepatitis B infection. Only three out of seventeen required treatment.

Regarding occupation and the risk of NSIs shown in [Table 5], anaesthesiology technicians had the greatest exposure risk when compared to office workers, followed by doctors, nurses, and housekeeping workers. Workers under twenty five years old, and those between twenty five and thirty five years old, were at increased risk for NSIs when compared to workers older than forty five years, [OR = 3.12,95% CI (1.19-8.19), *P* = 0.02] and [OR = 3.05,95% CI (1.42 - 6.57), *P* < 0.01] respectively, as shown in Table 6.

**Table 5:** Needlestick injuries risk estimate by type of work, using logistic regression

Occupation	OR	CI 95%	*P*
Nurses	6.75	1.56-29.03	0.01
Doctors	10	2.10-47.57	0.00
Housekeeping workers	5.33	1.20-23.61	0.02
Laboratory technicians	4.66	0.95-22.9	0.05
Anesthesiology technicians	16	2.55-100	0.00
Dialysis technicians	8	1.53-41.63	0.01
Office workers^R^	-	-	-

R Reference occupation group; OR = Odds ratio; CI 95% = Confidence interval 95%

**Table 6:** Needlestick injuries risk estimate by type of age group, using logistic regression

Age group (years)	OR	CI 95%	*P*
≤25	3.12	1.19-8.19	0.02
26-35	3.05	1.42-6.57	0.00
36-45	1.17	0.52-2.64	0.70
>45^R^	-	-	-

R Reference age group; OR = Odds ratio; CI 95% = Confidence interval 95%

Of the 321 HCWs, 165 (51.4%) had practiced for less than five years in the medical field. Our study shows that experienced staff (work duration more than five years), tend to have less NSIs annually compared to new staff (work duration less than five years). [OR = 0.54, 95% CI (0.32-0.92), *P* = 0.02].

## DISCUSSION

In the Middle East, the majority of HBV infections occur through childhood and perinatal transmission, yet there is limited information on sexual transmission in societies of the Middle East.[9] To decrease the prevalence of HBV infection, many countries introduced Hepatitis B vaccination through their expanded program on immunization (EPI). In Syria Hepatitis B vaccination was added to the EPI program in 1991. Data from the Syrian Ministry of Health in 2004, shows that the overall prevalence of HBV is 5.62%, with Aleppo having the highest infection rate among all the Syrian provinces, with a rate of 10.6%.[10]

It is well known that vaccine-induced antibodies decline gradually over time, and as many as 60% of those who initially responded to vaccination will lose detectable antibodies in eight years. However, booster doses of vaccines are not recommended for immunocompetent HCWs, because persons who respond to the initial vaccine series remain protected against clinical hepatitis and chronic infection even when their anti-HBs levels become low or undetectable.[13–15] In our study of 180 workers, who previously received three doses of vaccination, 30 workers (16.7%) had anti HBs antibody titers of less than 100 IU/L, while nine had titers less than 10 IU/L.

In the Aleppo University Hospitals there are no designated trolleys, gloves, or disposal facilities at the bedside. HCWs frequently work alone, and patients are often unrestrained during these procedures. Sharps and needles used are either disposed in garbage collectors in the patient's rooms or carried back to the needle disposal box. With the huge workload and the absence of protective measures, HCWs at the Aleppo University Hospitals are exposed to a high risk of getting NSIs. Another dilemma that needs to be resolved is the underestimation of the importance of immediate NSI reporting among HCWs.

Underreporting of NSI is a common problem in our healthcare facilities. Although hospital employees are requested to report such accidents, a lot of injuries go unreported. One of the limitations that we had was the recall bias, which is why we reviewed each employee's medical charts and hospital injury reports. Nevertheless, some of these injuries were not previously reported.

To our knowledge, our study is the first to estimate the NSIs, HBV chronic infection, and vaccination status among HWCs in tertiary hospitals in Aleppo city and Syria. A previous biphasic study, the Focus Project, conducted in Syria from 2001 to 2004, was designed to follow-up on the effects of the implanted safety measures.[11] The study showed that 61% of HCWs got at least one NSI during a period of 12 months, in 2001, which decreased to 14% after the implementation of safety instructions and measurements, compared to 76.6% in our study. That study was performed mainly in the primary and secondary healthcare facilities, along with some private hospitals. Seventy percent of the facilities were utilized for immunization purposes, with a much lower workload than the tertiary hospitals in the Aleppo University. This could explain the difference in results between our study and the Focus Project. Another explanation may be that it was due to the safety measures themselves: In 2001, safety boxes were used in 63% of the vaccination areas and 22% of the curative injection areas in the Focus Project, while in 2004, safety boxes were available in almost all healthcare facility departments where injections were given. In 2004, however, safety boxes were regularly supplied to only 13% of the hospitals. Consequently, sharps exposing staff to the risk of NSIs were found in open containers in 90% of the hospitals, two-handed recapping was performed in 76% of the hospitals, and NSIs were reported by 45% of HCWs in hospitals versus 14% of HCWs in other health facilities. These data are compatible with our results and highlight the fact that an increased workload leads to an increased chance of NSIs, due to the reluctance and delay in applying safety measures.

Another study,[9] conducted in 2001, shows that 76% of the Syrian general population previously received three doses of vaccination against HBV infection, while the percentage of complete vaccination among HCWs in our study is only 56.1%. In a study conducted in Turkey in 2005,[16] 68% of the HCWs were completely vaccinated, and doctors and nurses were at the same risk of NSIs (27% of the nurses and doctors had sustained NSIs during a period of six months). In our study, anesthesiology technicians had the highest risk of NSIs followed by doctors and then nurses. Twenty workers (6%) were HBsAg positive compared to nine HCWs (2.8%), in our study.

In several investigations of nosocomial hepatitis B outbreaks, most infected HCWs could not recall an overt percutaneous injury.[17,18] HBV infections in HCWs with no history of nonoccupational exposure or occupational percutaneous injury might have resulted from direct or indirect blood or body fluid exposures that inoculated HBV into cutaneous scratches, abrasions, burns, other lesions, or on mucosal surfaces.[19–21]

A study by Fisman *et al*.[22] showed that nurses report the most frequent exposures to NSIs (37.9%), followed by resident or fellow physicians (11.4%), attending physicians (10.7 %), surgeons (9.0%), phlebotomists (5.4%), and non-laboratory technologists (4.7%). In our study anesthesiology technicians had the highest rate and risk in acquiring both NSIs and HBV infection, while doctors ranked second in NSI exposure. This could be explained by the fact that the risk of HBV infection is primarily related to the degree of contact with blood in the work place and the increased risk of exposure during long work shifts, factors which are associated with a three-fold increase in the risk of NSI.[22]

In studies of HCWs who sustained injuries from needles contaminated with blood containing HBV,[23] the risk of developing clinical hepatitis if the blood was both HbsAg- and HBeAg-positive was 22-31%, and the risk of developing serologic evidence of HBV infection was 37-62%. In comparison, the risk of developing clinical hepatitis from a needle contaminated with HBsAg-positive and HBeAg-negative blood was 1-6%, and the risk of developing serologic evidence of HBV infection was 23-37%. Other studies found that as little as 10 ml[8] of HBV positive blood could transmit infection to a susceptible host.[24]

In some studies, the prevalence of HBV carriers in Syria varies from 3 to 5%,[24] while it ranges from 5 to 10% in others,[10] which highlights that HCWs are at an increased risk of exposure to patients with asymptomatic HBV infection. This fact should promote the necessity of having all HCWs vaccinated against HBV. Although the prevalence of HBV chronic infection in our study (2.8%) is less than in other studies conducted in Pakistan (7.5%) and other countries, it should still be noted that there are simple ways to prevent transmission, which should not be overlooked.[25] In 1991, OSHA required that all HCWs with reasonably anticipated exposure to blood be offered HBV vaccine. Studies suggest that this strategy has been highly successful in reducing HBV infection among HCWs with a 95% decline in the incidence of hepatitis B infection among American HCWs between 1983 and 1995.[26]

Another method to reduce exposure is warning signs. Where needles are used, existing policies on their use and disposal should be implemented and regularly reinforced to minimize risk to staff and patients. To be safe, HCWs must adhere to standard precautions and follow fundamental infection-control principles. In our study, housekeeping workers reported that they injured themselves with needles not disposed of properly; concealed in linen or regular garbage.

Preventing NSIs is the most effective way to protect workers from the infectious diseases that needlestick accidents transmit. Therefore, pre-employment education in health and safety must be a part of all courses for prospective healthcare workers. Thorough occupational health and safety practices must be promoted to all staff throughout their career, while awareness of occupational health and safety and its importance to their own health and well being should be stressed. This could be achieved by continuing training programs over time to ensure that HCWs are kept up-to-date and aware of new needlestick policies, practices, and procedures. A comprehensive NSI prevention program would include: Employee training, recommended guidelines, safe recapping procedures, effective disposal systems, surveillance programs, and improved equipment design.

## CONCLUSION

The hepatitis B virus is a global public health problem. In healthcare facilities, transmission generally occurs from patient to healthcare worker. However, with the implementation of routine hepatitis B vaccination and the use of standard precautions to prevent exposure to blood-borne pathogens, HBV infection in this population should become a rare occurrence. We highly encourage the usage of disposable needles and equipment, proper sterilization of surgical instruments, enforcement of infection control measures and vaccination of healthcare workers.

Although a high percentage of doctors, nurses, and other technicians have been fully vaccinated against hepatitis B, efforts need to be made to improve this coverage and to also provide vaccination to housekeeping workers who have an increased risk of acquiring a vaccine preventable disease.
